# Minimizing human interference in an online fully automated daily adaptive radiotherapy workflow for bladder cancer

**DOI:** 10.1186/s13014-024-02526-2

**Published:** 2024-10-07

**Authors:** Sana Azzarouali, Karin Goudschaal, Jorrit Visser, Laurien Daniëls, Arjan Bel, Duncan den Boer

**Affiliations:** 1 Radiation Oncology, Amsterdam UMC location Vrije Universiteit Amsterdam, De Boelelaan 1117, Amsterdam, The Netherlands; 2 Cancer Center Amsterdam, Cancer Therapy, Treatment and quality of life, Amsterdam, The Netherlands; 3 Radiation Oncology, Amsterdam UMC location University of Amsterdam, Meibergdreef 9, Amsterdam, The Netherlands

**Keywords:** Online adaptive radiotherapy, Bladder cancer, Automation, Fiducial markers, Reoptimization, Artificial intelligence, Radiotherapy, CBCT, Focal boost

## Abstract

**Purpose:**

The aim was to study the potential for an online fully automated daily adaptive radiotherapy (RT) workflow for bladder cancer, employing a focal boost and fiducial markers. The study focused on comparing the geometric and dosimetric aspects between the simulated automated online adaptive RT (oART) workflow and the clinically performed workflow.

**Methods:**

Seventeen patients with muscle-invasive bladder cancer were treated with daily Cone Beam CT (CBCT)-guided oART. The bladder and pelvic lymph nodes (CTV_elective_) received a total dose of 40 Gy in 20 fractions and the tumor bed received an additional simultaneously integrated boost (SIB) of 15 Gy (CTV_boost_). During the online sessions a CBCT was acquired and used as input for the AI-network to automatically delineate the bladder and rectum, i.e. influencers. These influencers were employed to guide the algorithm utilized in the delineation process of the target. Manual adjustments to the generated contours are common during this clinical workflow prior to plan reoptimization and RT delivery. To study the potential for an online fully automated workflow, the oART workflow was repeated in a simulation environment without manual adjustments. A comparison was made between the clinical and automatic contours and between the treatment plans optimized on these clinical (D_clin_) and automatic contours (D_auto_).

**Results:**

The bladder and rectum delineated by the AI-network differed from the clinical contours with a median Dice Similarity Coefficient of 0.99 and 0.92, a Mean Distance to Agreement of 1.9 mm and 1.3 mm and a relative volume of 100% and 95%, respectively. For the CTV_boost_ these differences were larger, namely 0.71, 7 mm and 78%. For the CTV_boost_ the median target coverage was 0.42% lower for D_auto_ compared to D_clin_. For CTV_elective_ this difference was 0.03%. The target coverage of D_auto_ met the clinical requirement of the CTV-coverage in 65% of the sessions for CTV_boost_ and 95% of the sessions for the CTV_elective_.

**Conclusions:**

While an online fully automated daily adaptive RT workflow shows promise for bladder treatment, its complexity becomes apparent when incorporating a focal boost, necessitating manual checks to prevent potential underdosage of the target.

**Supplementary Information:**

The online version contains supplementary material available at 10.1186/s13014-024-02526-2.

## Background

Bladder cancer is among the 10 most diagnosed cancers worldwide [1]. One out of five patients develops muscle-invasive bladder cancer which is known for its poor prognosis with a 5-year survival rate of 50% [2]. These patients can be treated either by radical cystectomy or by combining radiotherapy (RT) with chemotherapy and transurethral resection of the bladder tumor (TURBT).

Image-guided RT aims to deliver the planned dose in the right location. For bladder cancer this is complicated, however, as several organs in the pelvic region, for example the bladder and rectum, show daily variations in size, position and shape. To ensure target coverage in the presence of these variations larger margins are needed, resulting in a relatively large volume of irradiated surrounding healthy tissue [3, 4]. By treating with a focal boost, a higher dose is given to the tumor bed and a somewhat lower dose to the bladder (and elective pelvic lymph nodes) allowing for reduced toxicity [3, 4]. Fiducial markers have been shown to improve visibility of the tumor bed on CT and Cone Beam CT (CBCT) and guide the delivery of such an additional boost dose [5, 6].

Another method to obtain toxicity reduction while retaining target coverage, is online adaptive radiotherapy (oART). By acquiring an image at the start of every treatment session, either by CBCT or MRI, the treatment plan can be reoptimized while taking into account the daily, interfraction anatomical variations. Both CBCT- and MR-guided oART have been reported to be feasible for the treatment of patients with bladder cancer [6–10]. However, studies on CBCT-guided oART [6, 7] reported more conformity and a lower on-couch time compared to studies on MR-guided oART [8, 9].

To deal with the intrafraction variations a short on-couch time (duration of the patient lying on the table) is important as this leads to less bladder filling and smaller corresponding planning target volume (PTV) margins. Average filling rates between 1 and 4 ml/min have been reported [11]. A shorter on-couch time also increases patient comfort by decreasing the time the patients have to lie still and retain urine in their bladder.

Delineation of the daily anatomy can be labor intensive and time consuming. Automating this process could alleviate both of these challenges. The image quality of CBCT has shown to be sufficient to apply automatic bladder segmentation [12, 13]. Accurate automatic delineations can be expected to be consistent, leading to a situation where the treatment quality would be less dependent on staff experience and human interobserver variations [14].

A fast workflow for the treatment of bladder cancer would combine CBCT-guided oART with automatic segmentation. Such a workflow, employing artificial intelligence (AI) to automatically delineate the bladder and rectum has been demonstrated to be feasible for bladder cancer treatment [6, 7]. The bladder and rectum delineations have a large influence on the shape and position of the targets and are referred to as “influencers”. These influencers are used to guide the propagation of the target and organs-at-risk (OARs). However, in previous work we showed that for the case of bladder cancer, most of the sessions currently include manual corrections to the target delineations leading to an increased on-couch time of 5 min [6]. These 5 min correspond to an additional necessary margin up to 3 mm [15, 16].

Our aim was to study the potential of a fully automated AI-driven CBCT-guided oART workflow, without online manual corrections, for bladder cancer employing a focal boost. The evaluation was done by a geometric and dosimetric comparison between the simulated automated oART workflow and the clinically performed oART workflow.

## Methods

### Patient characteristics and the clinical workflow

Between April 2021 and August 2023, 17 patients with muscle-invasive bladder cancer (see also Additional file 1) were treated with AI-driven CBCT-guided oART (Ethos Therapy™, version 1.1, Varian a Siemens Healthineers Company, USA). Within 6 weeks after TURBT the patients underwent chemotherapy (Mitomycin-c/ Capecitabine) starting on the first day of RT treatment.

RT was given over a period of 4 weeks with a total of 20 fractions. The clinical target volume (CTV_elective_) was defined as the pelvic lymph nodes (internal iliac, obturator, hypogastric and perivesical until lower part of sacroiliac joint), urethra (men: 2 cm proximal, women: 1 cm proximal) and whole bladder. This CTV_elective_ received a total dose of 40 Gy (including positive lymph nodes when present). The (remnant) tumor or resection scar, which for simplicity both will be referred to as gross tumor volume (GTV), received an additional boost dose of 15 Gy given as a simultaneous integrated boost (SIB). All structures were manually delineated on a reference CT. CTV_boost_ was defined as the GTV with an isotropic 5 mm margin. The PTV margin for the CTV_boost_ was also 5 mm and a minimum of 7 mm was used for the PTV margin of the bladder based on the bladder filling observed during pretreatment (using 2 planning CTs). More details about the pretreatment (including placement of fiducial markers, treatment planning constraints, margins and delineation of target and OARs) were previously reported and are available online [6].

The online adaptive session started by acquiring a CBCT at the start of the daily anatomy. The AI network (vendor supplied) used this CBCT as input to automatically delineate the bladder and rectum (for more details on the AI network and automatic segmentation see [17]). In the clinical workflow, manual adjustments to these delineations were allowed (3 physicians and 9 radiation therapists were involved). Subsequently, a deformable registration was performed from the reference CT to the CBCT, used to generate a synthetic CT for dose calculation. The bladder and rectum influence the position and shape of the GTV, therefore the software used these to guide the deformable registration to come to a GTV delineation for a manual check. OARs (small bowel, bowel bag, sigmoid, left and right femur head) were propagated using deformable registration from the reference CT to the daily image. An adaptive plan was generated taking into account the daily anatomy (see also [6] for more details of the oART workflow).

### Simulation of an automated oART workflow

**Fig. 1:**
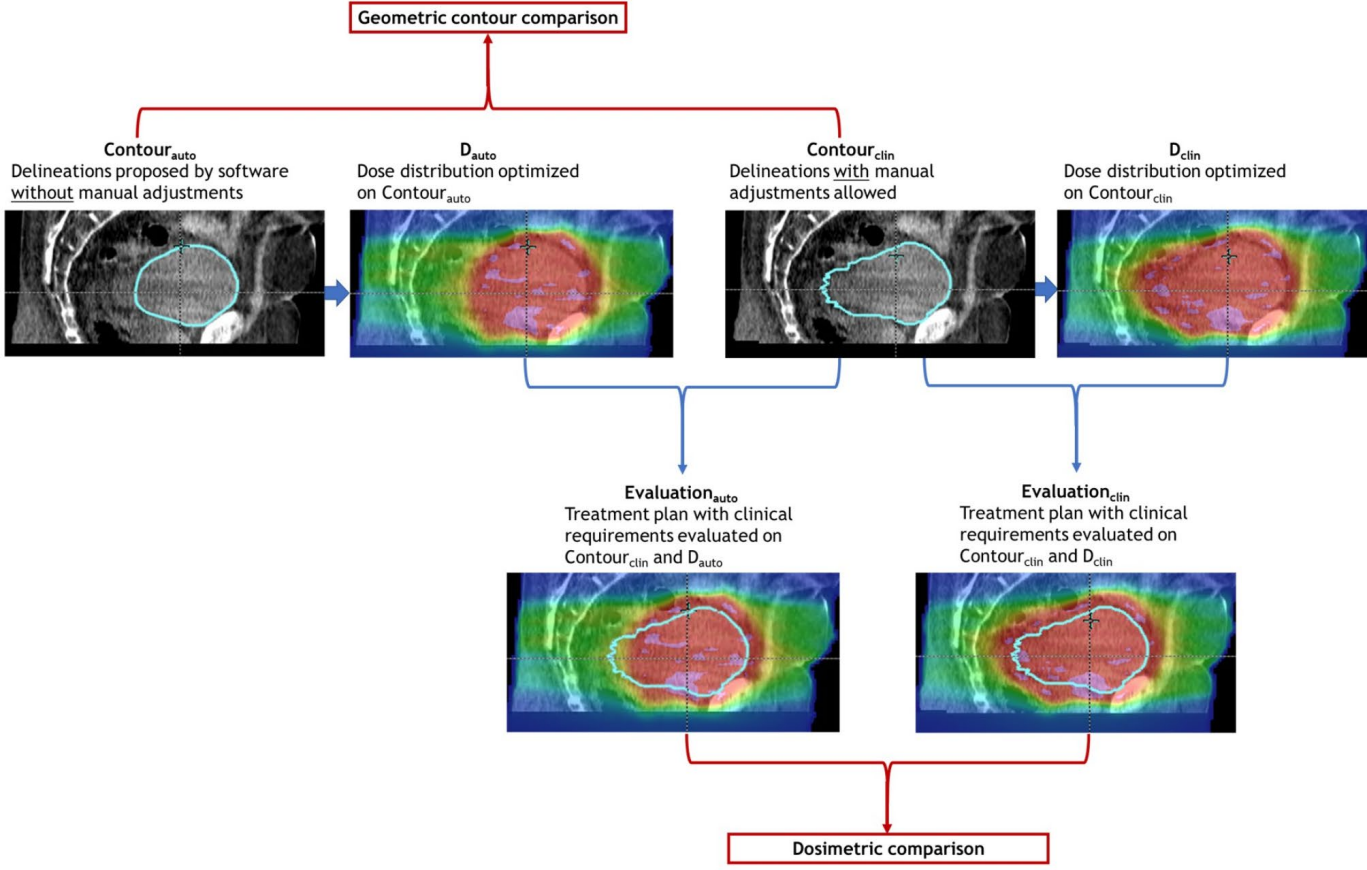
Evaluation of the treatment plan from the online fully automated daily adaptive workflow as compared to the clinically used treatment plan

To study the potential of an oART workflow that would be fully automated during the online sessions, two data sets were compared (see Fig. 1). The first data set consists of 340 online reoptimized treatment plans from the 17 patients (20 fractions per patient) treated in the clinic as described in the previous paragraph (Evaluation_clin_). Evaluation_clin_ consists of the structure set (Contour_clin_) and the dose distribution (D_clin_) extracted from the online fractions that included manual adjustments to the delineations if deemed necessary. The second data set was obtained by first simulating the oART workflow steps on the same daily CBCTs (Ethos test environment, version 1.1, Varian a Siemens Healthineers Company, USA). In contrast to the clinical workflow, no manual adjustments were made to the structure set, including influencers and target, automatically proposed by the software (Contour_auto_). A geometric evaluation was done by comparing Contour_clin_ with Contour_auto_ as described in the next paragraph. To also evaluate the dosimetric effects, a dose distribution was generated by performing a reoptimization based on Contour_auto_ (D_auto_). To evaluate if the simulated dose distribution would have led to acceptable treatments, the dose-volume histogram of Contour_clin_ was calculated using D_auto_ (Evaluation_auto_), where Contour_clin_ was used as the ground truth. For this evaluation, the clinical requirements (for details see previous work) were assessed [6].

### Workflow comparison

We first monitored the number of manual corrections that were applied to the influencers (i.e. bladder and rectum) and GTV in the clinical workflow. To compare the online fully automated workflow with the clinical workflow, all 20 sessions from each of the 17 patients were included for evaluation. The evaluation consisted of a geometric contour comparison between Contour_auto_ and Contour_clin_, a dosimetric comparison between D_auto_ and D_clin_ and an analysis of what might influence the accuracy of the online fully automated workflow. All metrics were extracted using home built software (Matlab R2021a, Mathworks).

### Geometric contour comparison

To analyze the geometric differences between Contour_auto_ and Contour_clin_, the Dice Similarity Coefficient (DSC), the relative volume, the 95-percentile Hausdorff Distance (95%HD) and the Mean Distance to Agreement (MDA) were extracted from each of the 340 session for the influencers and the CTV_boost_ [18–20]. The relative volume was defined as V_auto_/V_clin_, where V_auto_ represents the volume of Contour_auto_ and V_clin_ the volume of Contour_clin_.

### Dosimetric and statistical analysis

A dosimetric evaluation of the online fully automated workflow was done by evaluating Contour_clin_ of the GTV, CTV_boost_, CTV_elective_, the planning target volume (PTV) surrounding CTV_boost_ (PTV_boost_) and the PTV surrounding CTV_elective_ (PTV_elective_) in dose distributions D_clin_ and D_auto_. The target coverage of these clinical contours was determined for the two dose distributions by extracting the volume of these structures receiving a minimum of 95% of the prescribed dose (V_95%_). The clinical requirement for the V_95%_ was a minimum of 98% [6]. To get an indication of the healthy tissue sparing, the V_95%_ outside the previously mentioned target structures (V_95%,out_) was obtained by:

$$\:{V}_{95\%,\:\:out}=\:{V}_{95\%,\:\:body}-\:{V}_{95\%},$$

where V_95%,body_ represents the total volume of the body receiving a minimum of 95% of the prescribed dose per fraction [6]. To compare the difference between D_auto_ and D_clin_ using these metrics, a statistical analysis was done by a paired Wilcoxon signed-rank test. A Bonferroni corrected significance level of 0.5% was used. Besides performing a dosimetric comparison between D_auto_ and D_clin_, we also tested whether the target coverage achieved with D_auto_ would have met the clinical requirements.

### Volume differences and delineation accuracy

To get an insight of what might influence the software delineation accuracy, the variations in bladder and rectum volume were analyzed. Since the influencers guide the online GTV delineation, the volume difference between these influencers on the reference CT and the online CBCT was extracted to determine its effect on the CTV_boost_ coverage (V_95%_). The number of sessions meeting the clinical requirement for the target coverage was evaluated by using volume ranges of 50 cm^3^ and 25 cm^3^ for the volume differences of the bladder and rectum, respectively. To complete the evaluation, the effect of this volume difference on the target coverage of CTV_elective_ was also analyzed.

## Results

In the clinical workflow, the delineations proposed by the AI network were manually corrected for the bladder and rectum, in 91% and 13% of the sessions, respectively. The proposed GTV delineation was corrected in 68% of the sessions. In only 4% of the sessions no manual adjustments were made to any of the delineations.

### Geometric contour comparison

**Fig. 2:**
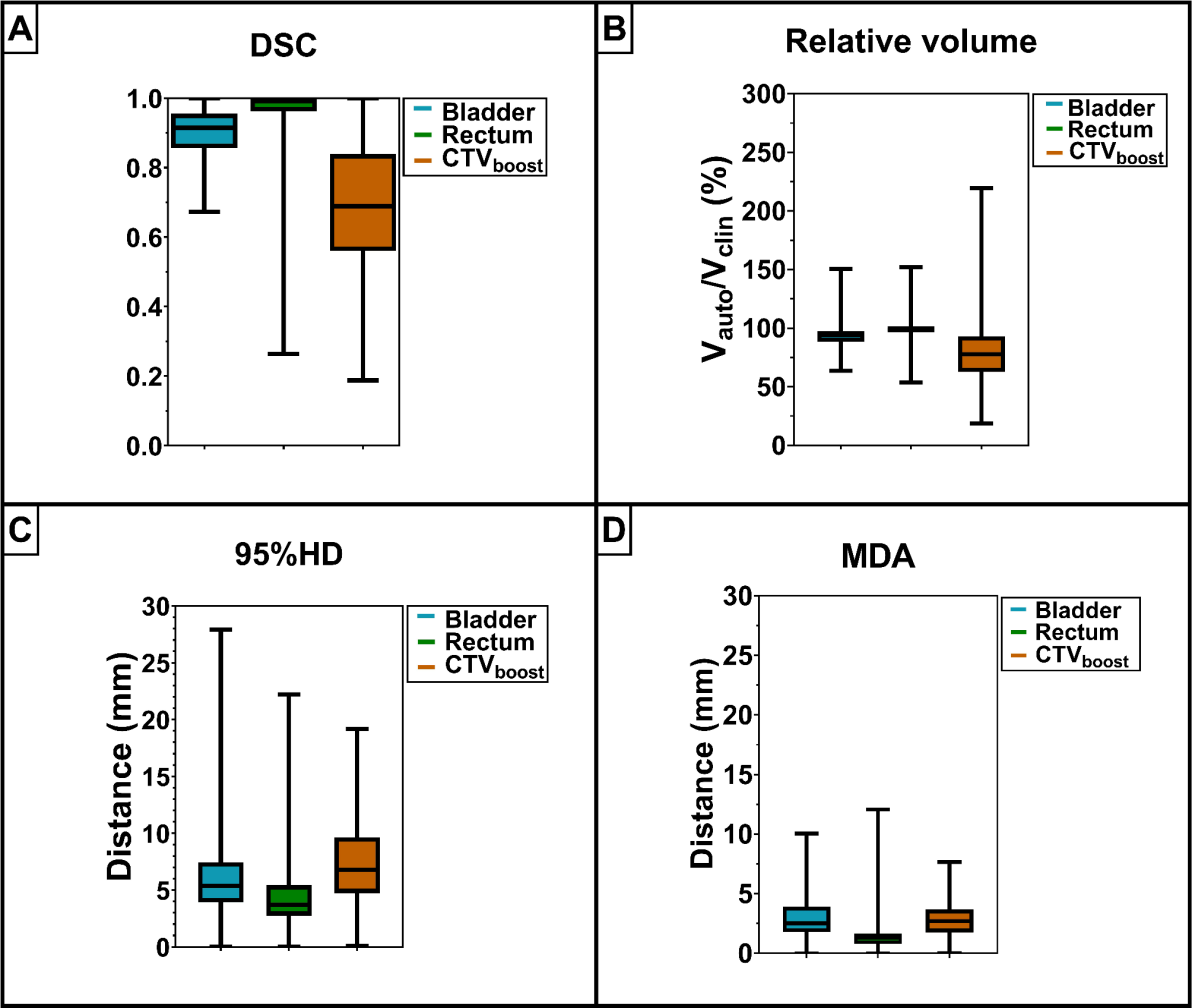
Geometric comparison of Contour_clin_ and Contour_auto_ for the rectum, bladder and the CTV_boost_ (*n* = 340 sessions). The boxplots of the Dice Similarity Coefficient (**A**), relative volume (**B**), 95% Hausdorff Distance (**C**) and Mean Distance to Agreement (**D**) represent the 1st and 3rd quartile with the median indicated inside and the whiskers representing the range

For all sessions of the complete patient group, the median DSC between Contour_auto_ and Contour_clin_ was 0.71 [0.19-1], 0.99 [0.26-1] and 0.92 [0.67-1] for the CTV_boost_, rectum and bladder, respectively (Fig. 2). These two contours differed with a median 95%HD of 7 [0–19] mm for the CTV_boost_, 4 [0–22] mm for the rectum and 5 [0–28] mm for bladder. The median relative volume (V_auto_/V_clin_) was 78% [19-220%] for the CTV_boost_, 100% [54-152%] for the rectum and 92% [64-150%] for the bladder. The MDA of the AI network was given by 1.9 [0-5.8] mm for the bladder and 1.3 [0-7.8] mm for the rectum. For the CTV_boost_ the MDA was given by a median of 2.3 [0-8.7] mm.

### Dosimetric analysis

**Fig. 3:**
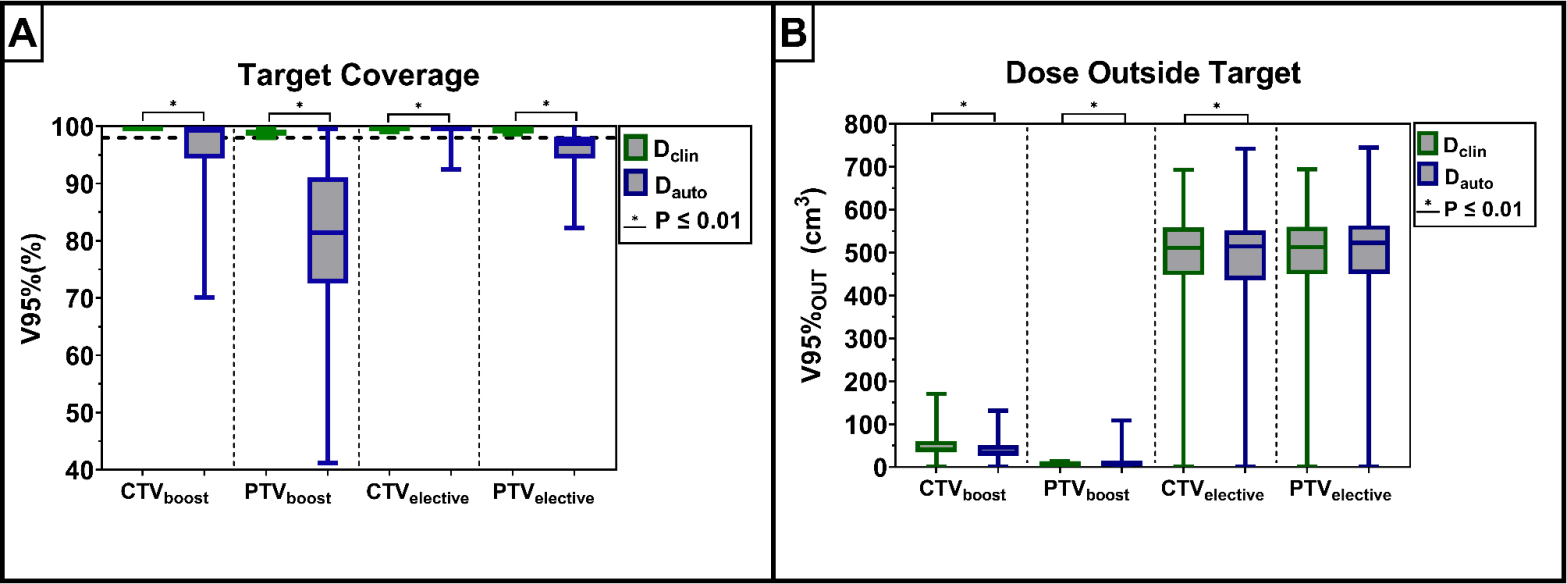
Dosimetric comparison of the target coverage (**A**) and the dose outside the target (**B**) between D_auto_ and D_clin_ (*n* = 340 treatment sessions) on Contour_clin_. The boxplots represent the 1st and 3rd quartile with the median indicated inside and the whiskers representing the range

**Fig. 4:**
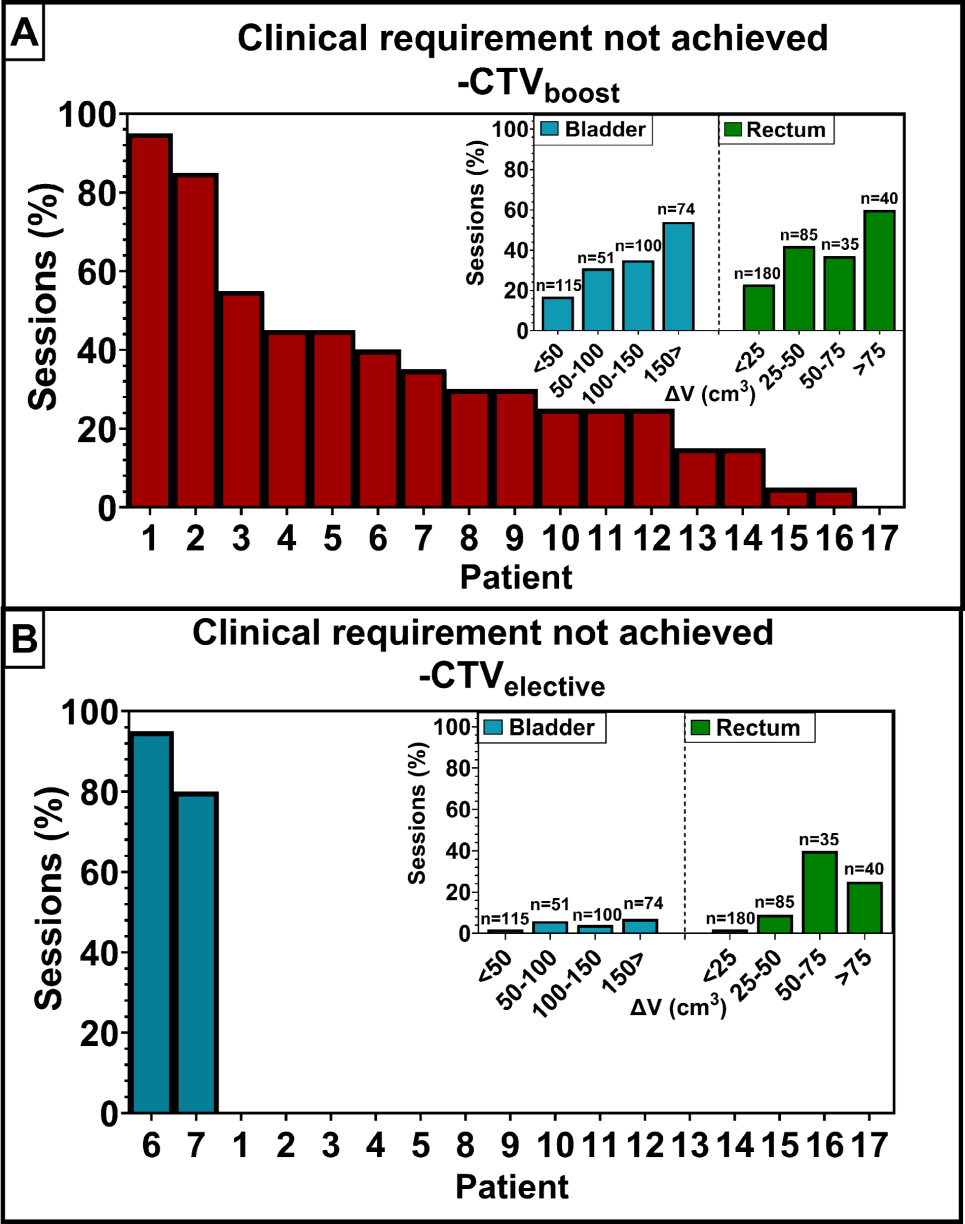
Percentage of sessions where D_auto_ would not have led to achievement of the clinical requirement for the target coverage (V_95%_ ≥ 98%) of Contour_clin_ per patient. The evaluation is done on Contour_clin_ for the CTV_boost_ (**A**) and CTV_elective_ (**B**). Insets: the percentage of sessions not meeting this clinical requirement per volume difference interval (see section “Volume differences and delineation accuracy” for more details). These volume differences were determined between the reference and online delineations of the influencers. The number above each bar represents the total number of sessions included in that specific volume difference interval

**Fig. 5:**
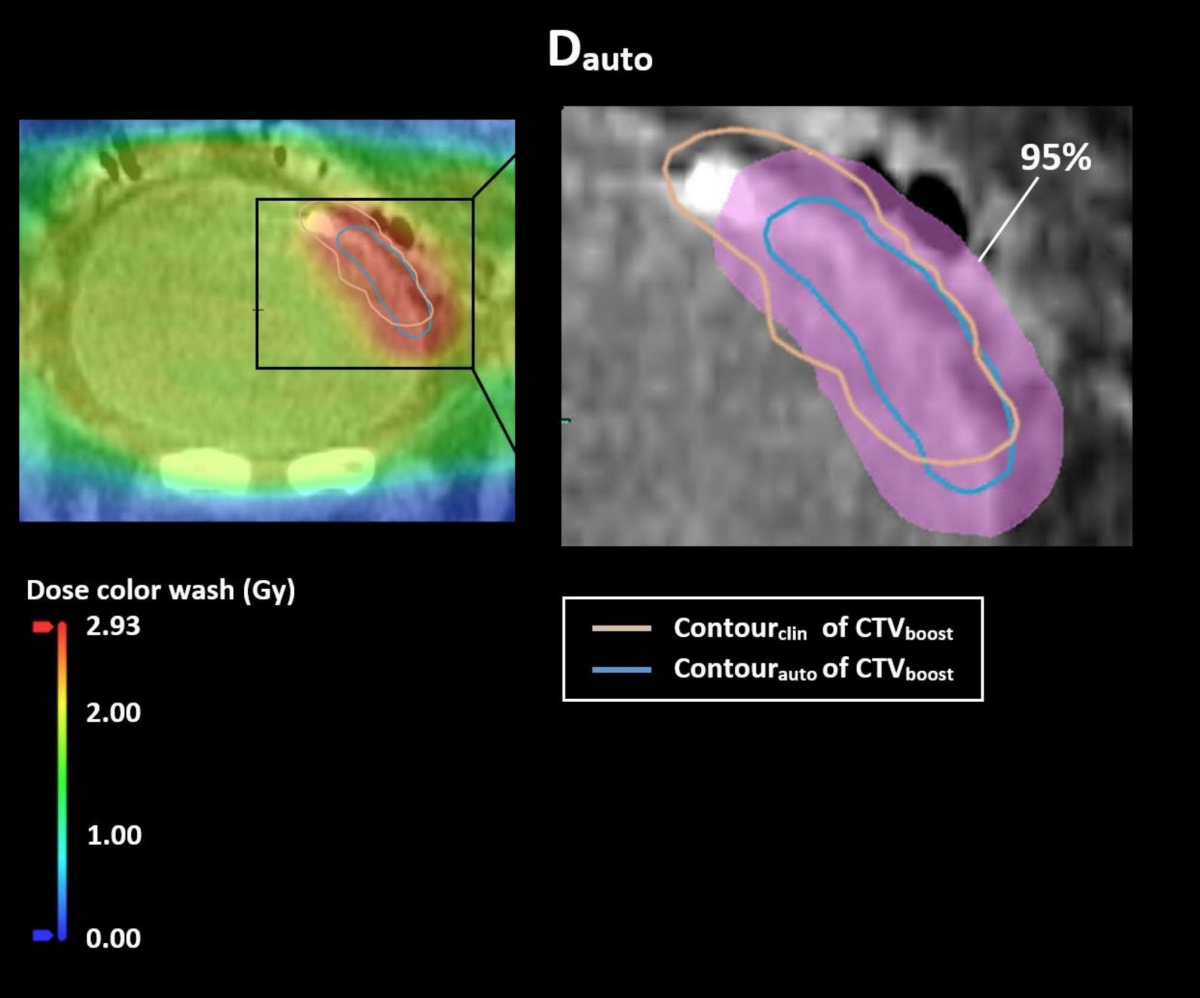
Example of a session not meeting the clinical requirements for the target coverage. D_auto_ is shown with both the automatically proposed CTV_boost_ delineation (Contour_auto_) and the manually adjusted CTV_boost_ delineation (Contour_clin_). The region receiving at least 95% of the prescribed dose is indicated (pink)

For the online fully automated workflow, D_auto_ resulted in the same median V_95%_ of the GTV as D_clin_ (see Additional file 2 for more details). For the CTV_boost_ and PTV_boost_ this median V_95%_ differed with 0.42% and 17.38%, respectively, resulting in less target coverage with D_auto_ (Fig. 3). Considering the CTV_elective_, the V_95%_ for D_auto_ and D_clin_ differed with a median of 0.03%, which was 2.44% for PTV_elective_. The target coverage, i.e. V_95%_, of D_auto_ was found to be statistically significantly different from D_clin_ for all of the previously mentioned target structures (boost and elective). This target coverage of D_auto_ met the clinical requirement of the CTV coverage in 65% of the sessions for CTV_boost_ and 95% of the sessions for the CTV_elective_ (Fig. 4). The remaining 5%, not meeting the CTV_elective_ coverage, was observed in two patients of which one had a diverticulum (see also Additional file 4). For the sessions with exclusively manual adjustments to the GTV-delineation, a median difference in CTV_boost_ coverage of 3% was observed between D_auto_ and D_clin_ (Additional file 3). For the PTV_boost_ this difference in target coverage was 22%. In Fig. 5 we can see an example of a session in which the clinical requirement for the CTV_boost_ coverage was not met.

The median volume outside the CTV_boost_ receiving a minimum of 95% of the prescribed dose, was 7.8 cm^3^ lower for D_auto_ compared to D_clin_. For PTV_boost_, CTV_elective_ and PTV_elective_ this difference was 2.7 cm^3^, 3.1 cm^3^ and 9.8 cm^3^, respectively. V_95%,out_ was for D_auto_ and D_clin_ always statistically significantly different except for the PTV_elective_.

### Volume differences and delineation accuracy

In 17% of the sessions in which the difference in bladder volume on the reference CT and online CBCT was smaller than 50 cm^3^, the CTV_boost_ coverage did not meet the clinical requirement (Fig. 4; see also Additional file 5 for more details). This requirement was not met in 54% of the cases, for sessions in which the bladder volume difference was larger than 150 cm^3^. Regarding the rectum, i.e. the other influencer, 23% of sessions failed to meet the clinical requirement for the CTV_boost_ coverage when the volume difference was small (< 25 cm3) and this percentage increased to 60% for sessions with a large volume difference (> 75 cm3).

## Discussion

In this study we investigated if an online fully automated daily adaptive RT workflow would meet the clinical requirements for bladder cancer patients treated with a SIB. Removing the need for manual contour adjustments would improve the efficiency of the oART workflow and potentially decrease the necessary margins to compensate for intrafraction bladder filling, due to shorter on-couch time. Our results showed that the median difference in target coverage between the automated sessions and clinical sessions was small (< 0.5%) and about two thirds of the sessions met the clinical requirements. Even though the median target coverage was similar, there is a large range for the coverage of the CTV with values as low as 70% for the CTV_boost_. These values are too low to be clinically acceptable.

The CTV_boost_ is delineated by the software based on an influencer guided deformable registration between the reference CT and online CBCT. If these influencers showed a larger volume difference between the reference CT and online CBCT, less of the sessions met the clinical requirements. This emphasizes the importance of methods that aim for lower variations in these volume differences, e.g. drink instruction, even while applying daily oART. Performing a couch shift prior to RT would correct for software delineation inaccuracy related to CTV_boost_ positioning, nevertheless, merely offering a partial solution given the automatic delineation volume being typically too small. To correct for this difference in size, the software might benefit from a marker guided delineation process. Fiducial markers are clearly visible on the CBCTs and are also used in clinical practice by the medical staff to localize the CTV_boost_ [6]. Another interesting alternative might be to train an AI network to delineate the CTV_boost_. However, this would require a lot of data from bladder cancer patients with fiducial markers implanted. With respect to CTV_elective_, the previously mentioned volume effect of the influencers on the CTV coverage was not observed. This can be explained by the fact that the bladder is delineated by the AI network using the online CBCT, without the reference CT as input. For one patient a diventriculum was present resulting in poor bladder delineations proposed by the AI network. Including more patients with a diventriculum in the training data set might increase the performance of the AI regarding this matter. More than 90% of the online fully automated daily adaptive RT sessions met the clinical requirements for CTV_elective_. This illustrates the potential for this workflow for whole bladder RT, when the focal boost is not applied [7].

Not requiring manual adjustments to the structure delineations would allow for a median time reduction of 5 min, i.e. 23% of the total on-couch time [6]. Taking into account the bladder filling during this time frame would mean 5–20 mL less volume increase and might result in a smaller displacement of the bladder wall of about 3 mm [11, 15, 16]. These values illustrate how a quicker workflow can lead to smaller PTV margins.

Besides the bladder filling, human interobserver variation is another factor causing an inaccuracy for the treatment of bladder cancer. Meijer et al. reported an interobserver variation up to 3 mm for the bladder [21]. The MDA of the bladder delineated by the AI network was within this range for 75% of the sessions. The MDA of the rectum was within the range reported for human interobserver variation (up to 5 mm) for 92% of the cases [22]. A limitation of our study was that the reference plan was based on manual delineations on the reference CT due to limitations of the current version of the software not allowing for automatic delineations on the reference CT.

The sessions not meeting the requirements for CTV_elective_ were concentrated within two patients, while in the other patients the CTV_elective_ coverage was adequate for all sessions. For the CTV_boost_ these suboptimal sessions were spread out over all patients. For a whole bladder treatment, the results suggest that if one could predict which patients are the ones where the automatic delineations are adequate, these patients could benefit from this online fully automated workflow. This could open up a strategy in which the first treatment sessions might predict whether the patient could be treated with a fully automated oART workflow for whole bladder treatments or if more manual adaptations of the bladder structure would be expected.

A study of Shelley et al. showed the median geometric differences, between the automatic and manually adjusted delineation, to be small for the bladder which was in line with our study [23]. However, our study showed that these small geometric differences can translate into clinically unacceptable dosimetric differences with respect to the CTV_boost_ coverage. Geometric differences in the influencer delineation would not only affect the CTV_elective_ delineation but also the CTV_boost_ delineation due to the influencer guided deformable registration. A semi-automated workflow, in which the influencers were corrected but the GTV-delineations were not, showed an improvement of 2% more median target coverage for the CTV_boost_ compared to the fully automated workflow in this study [6]. This shows that with the current technical capabilities, human interference and monitoring during the oART workflow for bladder cancer is still important in the case when a focal boost is included.

## Conclusion

An online fully automated daily adaptive RT workflow is promising for bladder treatments. However, in more complex situations as with a focal boost, the current implementation is inadequate. Manual checks remain important to mitigate the risk of target underdosage.

## Electronic supplementary material

Below is the link to the electronic supplementary material.

Additional file 1: Sex, age and tumor stage of patients included in this study

Additional file 2: Dosimetric comparison of the target coverage between D_auto_ and D_clin_ on Contour_clin_ including the GTV

Additional file 3: Difference in target coverage between D_auto_ and D_clin_ for sessions in which the GTV-delineation proposed by the software was manually adjusted

Additional file 4: Examples of sessions in which the AI delineation would have resulted in a CTV_elective_ coverage not meeting the clinical requirement

Additional file 5: Target coverage with automated software delineation versus bladder volume differences

## Data Availability

The datasets used and/or analyzed during the current study are available from the corresponding author on reasonable request.

## Abbreviations

AI: Artificial intelligence
CBCT: Cone Beam CT
MRI: Magnetic resonance imaging
CT: Computed tomography
CTV: Clinical target volume
D: Dose distribution
DSC: Dice Similarity Coefficient
Gy: Gray
GTV: Gross tumor volume
95%HD: 95%-percentile Hausdorff Distance
MDA: Mean Distance to Agreement
MRI: Magnetic resonance imaging
OAR: Organ-at-risk
oART: Online adaptive radiotherapy
PTV: Planning target volume
RT: Radiotherapy
SIB: Simultaneously integrated boost
sCT: Synthetic CT
TURBT: Transurethral resection of bladder tumor
V_95%_: Volume receiving a minimum of 95% of the prescribed dose

## References

[1] Sung H, Ferlay J, Siegel RL, Laversanne M, Soerjomataram I, Jemal A, et al. Global Cancer statistics 2020: GLOBOCAN estimates of incidence and Mortality Worldwide for 36 cancers in 185 countries. CA Cancer J Clin. 2021;71(3):209–49. 33538338 10.3322/caac.21660

[2] Knowles MA, Hurst CD. Molecular biology of bladder cancer: new insights into pathogenesis and clinical diversity. Nat Rev Cancer. 2015;15(1):25–41. 25533674 10.1038/nrc3817

[3] Lutkenhaus LJ, van Os RM, Bel A, Hulshof MCCM. Clinical results of conformal versus intensity-modulated radiotherapy using a focal simultaneous boost for muscle-invasive bladder cancer in elderly or medically unfit patients. Radiat Oncol. 2016;11(1):45. 26993980 10.1186/s13014-016-0618-6 PMC4797227

[4] Piet AHM, Hulshof MCCM, Pieters BR, Pos FJ, de Reijke TM, Koning CCE. Clinical results of a concomitant Boost Radiotherapy technique for muscle-invasive bladder Cancer. Strahlentherapie Und Onkol. 2008;184(6):313–8. 10.1007/s00066-008-1797-3 18535807

[5] de Ridder M, Gerbrandy LC, de Reijke TM, Hinnen KA, Hulshof MCCM. BioXmark^®^ liquid fiducial markers for image-guided radiotherapy in muscle invasive bladder cancer: a safety and performance trial. Br J Radiol. 2020;93(1111):20200241. 32463291 10.1259/bjr.20200241 PMC7336065

[6] Azzarouali S, Goudschaal K, Visser J, Hulshof M, Admiraal M, van Wieringen N, et al. Online adaptive radiotherapy for bladder cancer using a simultaneous integrated boost and fiducial markers. Radiat Oncol. 2023;18(1):165. 37803392 10.1186/s13014-023-02348-8 PMC10557331

[7] Åström LM, Behrens CP, Calmels L, Sjöström D, Geertsen P, Mouritsen LS, et al. Online adaptive radiotherapy of urinary bladder cancer with full re-optimization to the anatomy of the day: initial experience and dosimetric benefits. Radiother Oncol. 2022;171:37–42. 35358605 10.1016/j.radonc.2022.03.014

[8] Mitchell A, Ingle M, Smith G, Chick J, Diamantopoulos S, Goodwin E, et al. Feasibility of tumour-focused adaptive radiotherapy for bladder cancer on the MR-linac. Clin Transl Radiat Oncol. 2022;35:27–32. 35571274 10.1016/j.ctro.2022.04.008 PMC9092067

[9] Hunt A, Hanson I, Dunlop A, Barnes H, Bower L, Chick J, et al. Feasibility of magnetic resonance guided radiotherapy for the treatment of bladder cancer. Clin Transl Radiat Oncol. 2020;25:46–51. 33015380 10.1016/j.ctro.2020.09.002 PMC7522378

[10] Sibolt P, Andersson LM, Calmels L, Sjöström D, Bjelkengren U, Geertsen P, et al. Clinical implementation of artificial intelligence-driven cone-beam computed tomography-guided online adaptive radiotherapy in the pelvic region. Phys Imaging Radiat Oncol. 2021;17:1–7. 33898770 10.1016/j.phro.2020.12.004 PMC8057957

[11] Kong V, Hansen VN, Hafeez S. Image-guided adaptive radiotherapy for bladder Cancer. Clin Oncol. 2021;33(6):350–68. 10.1016/j.clon.2021.03.023 33972024

[12] van de Schoot AJAJ, Schooneveldt G, Wognum S, Hoogeman MS, Chai X, Stalpers LJA, et al. Generic method for automatic bladder segmentation on cone beam CT using a patient-specific bladder shape model. Med Phys. 2014;41(3):31707. 10.1118/1.4865762 24593711

[13] Rosewall T, Xie J, Kong V, Bayley AJ, Chung P, Currie G, et al. Automated delineation of the normal urinary bladder on Planning CT and Cone Beam CT. J Med Imaging Radiat Sci. 2016;47(1):21–9. 31047160 10.1016/j.jmir.2015.09.011

[14] Huynh E, Hosny A, Guthier C, Bitterman DS, Petit SF, Haas-Kogan DA, et al. Artificial intelligence in radiation oncology. Nat Rev Clin Oncol. 2020;17(12):771–81. 32843739 10.1038/s41571-020-0417-8

[15] Khouya A, Pöttgen C, Hoffmann C, Ringbaek TP, Lübcke W, Indenkämpen F, et al. Adaptation time as a determinant of the Dosimetric effectiveness of online adaptive radiotherapy for bladder Cancer. Cancers. 2023;15(23):5629. 38067333 10.3390/cancers15235629 PMC10705074

[16] Wilson C, Moseshvili E, Tacey M, Quin I, Lawrentschuk N, Bolton D, et al. Assessment of Intrafraction Motion of the urinary bladder using magnetic resonance imaging (cineMRI). Clin Oncol. 2020;32(2):101–9. 10.1016/j.clon.2019.09.056 31607612

[17] Archambault Y, Boylan C, Bullock D, Morgas T, Peltola J, Ruokokoski E, et al. Making on-line adaptive radiotherapy possible using artificial intelligence and machine learning for efficient daily re-planning. Med Phys Int J. 2020;8:77–86.

[18] Crum WR, Camara O, Hill DLG. Generalized overlap measures for evaluation and validation in Medical Image Analysis. IEEE Trans Med Imaging. 2006;25(11):1451–61. 17117774 10.1109/TMI.2006.880587

[19] Taha AA, Hanbury A. Metrics for evaluating 3D medical image segmentation: analysis, selection, and tool. BMC Med Imaging. 2015;15(1):29. 26263899 10.1186/s12880-015-0068-x PMC4533825

[20] Jena R, Kirkby NF, Burton KE, Hoole ACF, Tan LT, Burnet NG. A novel algorithm for the morphometric assessment of radiotherapy treatment planning volumes. Br J Radiol. 2010;83(985):44–51. 19620177 10.1259/bjr/27674581 PMC3487247

[21] Meijer GJ, Rasch C, Remeijer P, Lebesque JV. Three-dimensional analysis of delineation errors, setup errors, and organ motion during radiotherapy of bladder cancer. Int J Radiat Oncol. 2003;55(5):1277–87. 10.1016/s0360-3016(02)04162-7 12654438

[22] Franco P, Arcadipane F, Trino E, Gallio E, Martini S, Iorio GC, et al. Variability of clinical target volume delineation for rectal cancer patients planned for neoadjuvant radiotherapy with the aid of the platform Anatom-e. Clin Transl Radiat Oncol. 2018;11:33–9. 29928706 10.1016/j.ctro.2018.06.002 PMC6008279

[23] Shelley CE, Bolt MA, Hollingdale R, Chadwick SJ, Barnard AP, Rashid M, et al. Implementing cone-beam computed tomography-guided online adaptive radiotherapy in cervical cancer. Clin Transl Radiat Oncol. 2023;40:100596. 36910024 10.1016/j.ctro.2023.100596 PMC9999162

