# Supplementary material for: Minimizing human interference in an online fully automated daily adaptive radiotherapy workflow for bladder cancer
Source: Radiat Oncol. 2024 Oct 7;19:138. doi: 10.1186/s13014-024-02526-2 (PMC11457325; doi:10.1186/s13014-024-02526-2)
Supplement: Supplementary file 1 — Additional file 1: Sex, age and tumor stage of patients included in this study [file 13014_2024_2526_MOESM1_ESM.pdf]

Patient characteristics

Patient	Sex	Age	Tumor stage
**1**	M	79	(cT2N0M0)G3
**2**	M	65	(cT2N0M0)G3
**3**	M	70	(cT3N0M0)G3
**4**	M	57	(cT3N0M0)G3
**5**	M	66	(pT1N0M0)G3
**6**	M	63	(cT3N0M0)G3
**7**	F	71	(cT2N0M0)G3
**8**	F	66	(cT2N0M0)G3
**9**	M	79	(cT2N0M0)G3
**10**	M	62	(cT2N0M0)G3
**11**	F	61	(pT2aN0M0)G3
**12**	M	82	(cT2N0M0)G3
**13**	F	78	(cT2N0M0)G3
**14**	M	49	(cT2N0M0)G3
**15**	M	66	(cT2N1M0)G3
**16**	M	76	(cT2N0M0)G3
**17**	M	63	(cT2N1M0)G3

Additional file 1 : Sex, age and tumor stage of patients included in this study.

Additional file 1. Minimizing human interference in an online fully automated daily adaptive radiotherapy workflow for bladder cancer. Sana Azzarouali, Karin Goudschaal, Jorrit Visser, Laurien Daniëls, Arjan Bel, Duncan den Boer.
