# Supplementary material for: Minimizing human interference in an online fully automated daily adaptive radiotherapy workflow for bladder cancer
Source: Radiat Oncol. 2024 Oct 7;19:138. doi: 10.1186/s13014-024-02526-2 (PMC11457325; doi:10.1186/s13014-024-02526-2)
Supplement: Supplementary file 2 — Additional file 2: Dosimetric comparison of the target coverage between Dauto and Dclin on Contourclin including the GTV [file 13014_2024_2526_MOESM2_ESM.pdf]

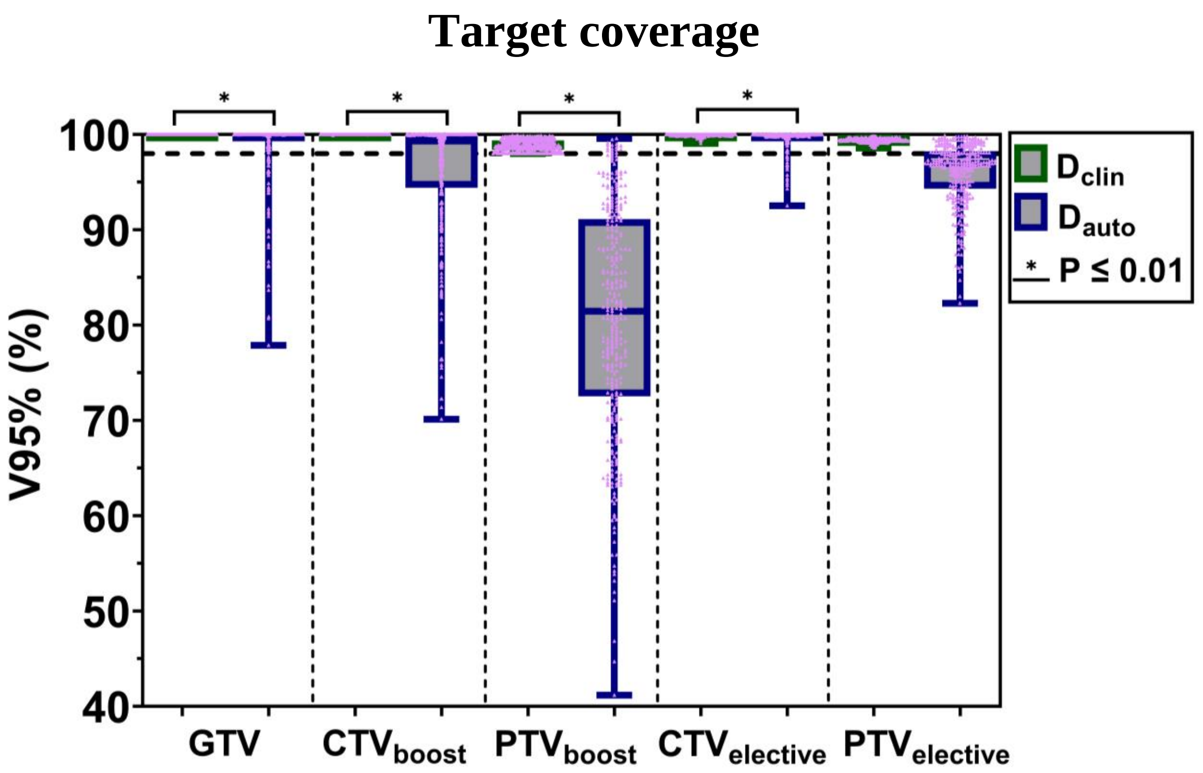

Additional file 2 : Dosimetric comparison of the target coverage between D_{auto} and D_{clin} (n = 340 treatment sessions) on $Contour_{clin}$ including the GTV. The boxplots represent the 1st and 3rd quartile with the median indicated inside and the whiskers representing the range. Individual data points are shown in pink.
