# Supplementary material for: Minimizing human interference in an online fully automated daily adaptive radiotherapy workflow for bladder cancer
Source: Radiat Oncol. 2024 Oct 7;19:138. doi: 10.1186/s13014-024-02526-2 (PMC11457325; doi:10.1186/s13014-024-02526-2)
Supplement: Supplementary file 3 — Additional file 3: Difference in target coverage between Dauto and Dclin for sessions in which the GTV-delineation proposed by the software was manually adjusted [file 13014_2024_2526_MOESM3_ESM.pdf]

Sessions with adapted GTV:
ΔTarget coverage

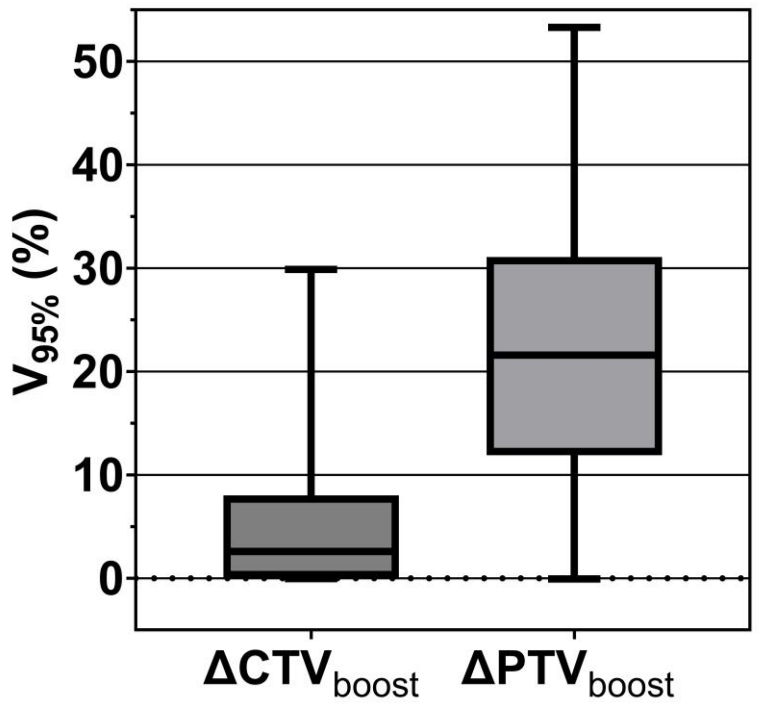

Additional file 3 : Difference in target coverage between D_{auto} and D_{clin} for sessions in which the GTV-delineation proposed by the software was manually adjusted (n = 231 sessions).

Additional file 3. Minimizing human interference in an online fully automated daily adaptive radiotherapy workflow for bladder cancer. Sana Azzarouali, Karin Goudschaal, Jorrit Visser, Laurien Daniëls, Arjan Bel, Duncan den Boer.
