# Supplementary material for: Minimizing human interference in an online fully automated daily adaptive radiotherapy workflow for bladder cancer
Source: Radiat Oncol. 2024 Oct 7;19:138. doi: 10.1186/s13014-024-02526-2 (PMC11457325; doi:10.1186/s13014-024-02526-2)
Supplement: Supplementary file 4 — Additional file 4: Examples of sessions in which the AI delineation would have resulted in a CTVelective coverage not meeting the clinical requirement [file 13014_2024_2526_MOESM4_ESM.pdf]

AI vs human bladder delineation: worst cases

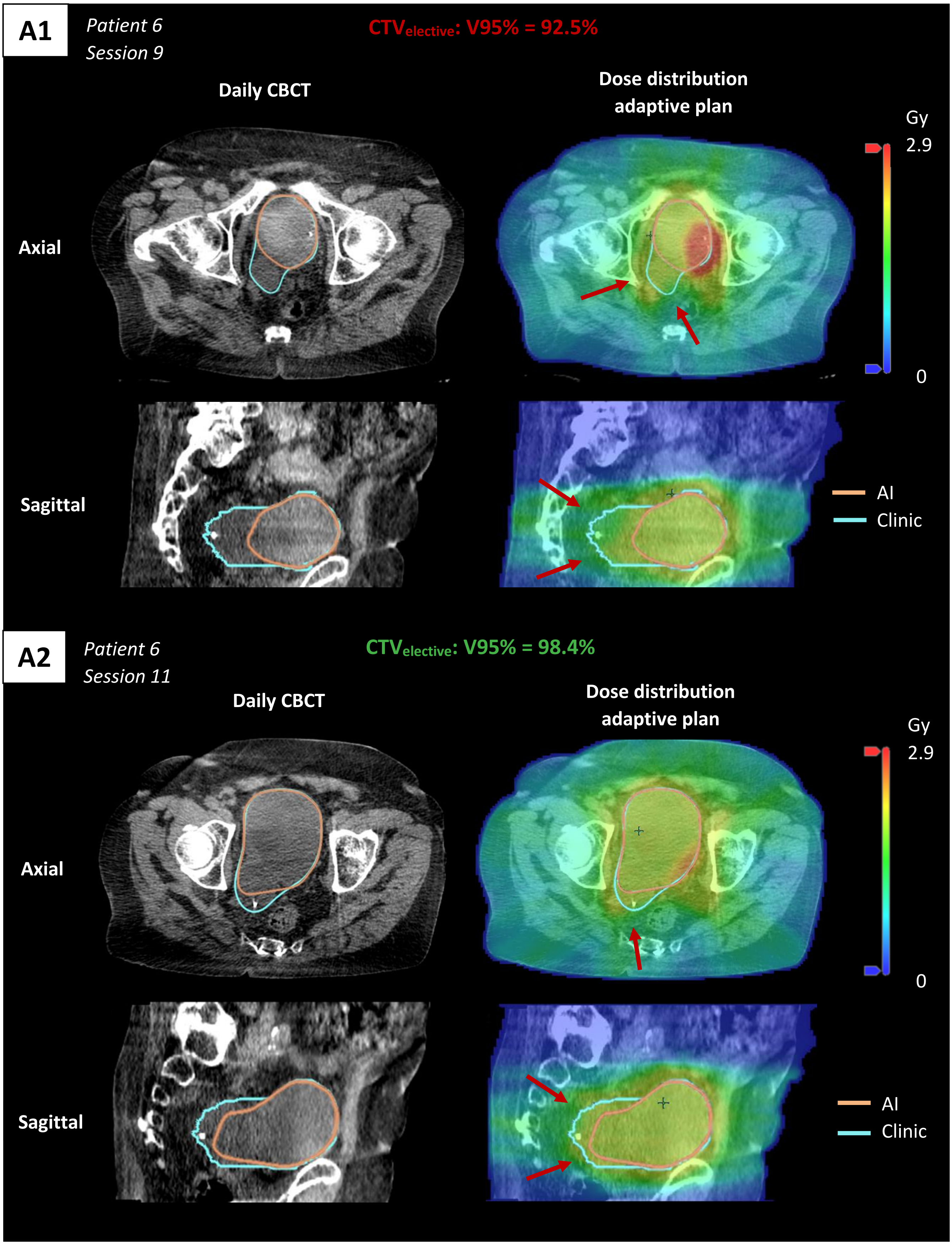

Additional file 4. Minimizing human interference in an online fully automated daily adaptive radiotherapy workflow for bladder cancer. Sana Azzarouali, Karin Goudschaal, Jorrit Visser, Laurien Daniëls, Arjan Bel, Duncan den Boer.

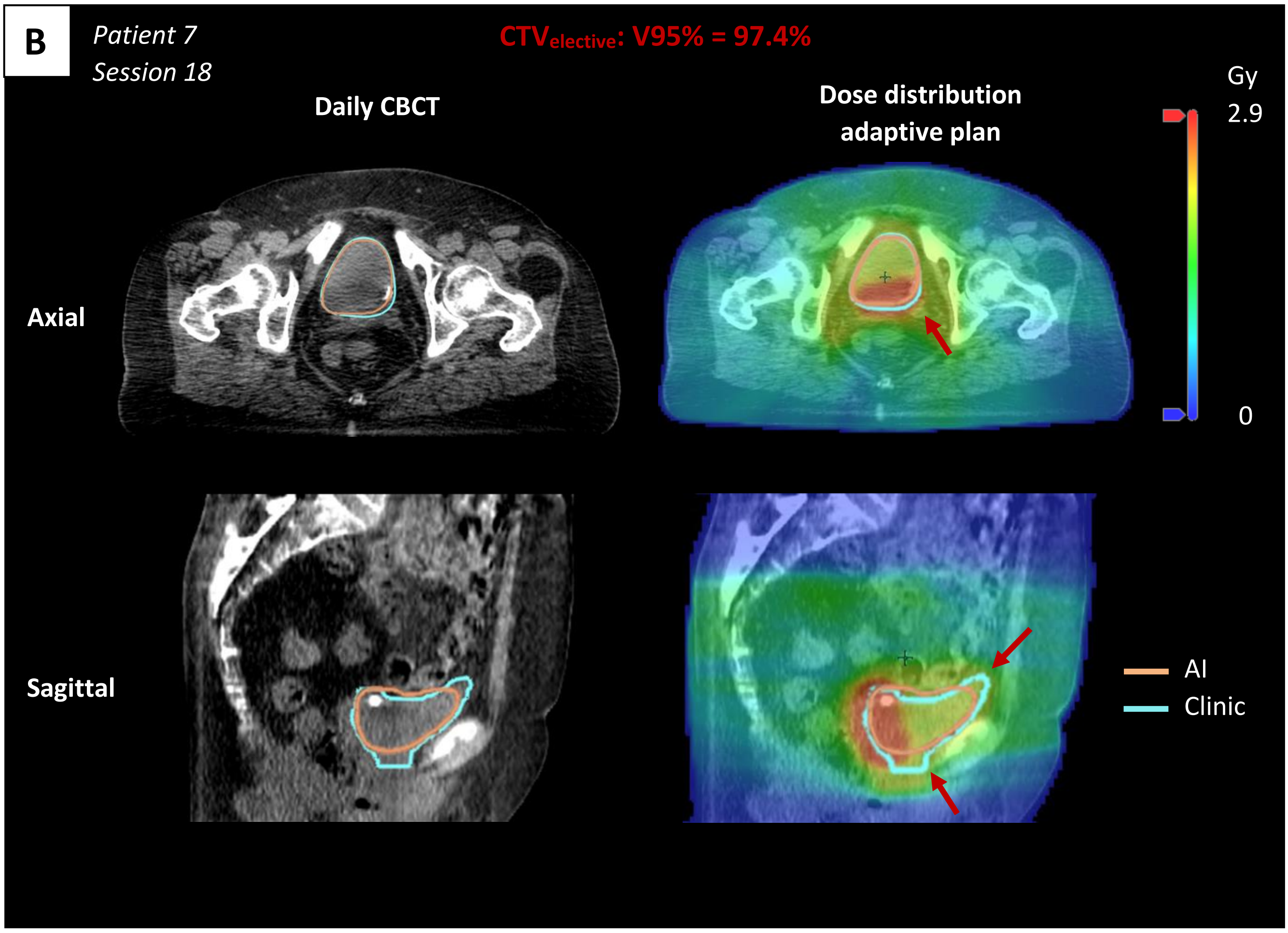

*Additional file 4 : Sessions in which the bladder delineation of the AI (orange) differed from the clinical (manually adjusted) bladder delineation (blue) on the daily CBCT with the dose distribution of the adaptive plan made on the AI delineations. Example of a session in which the AI delineation would have resulted in a $CTV_{elective}$ coverage (difference in target coverage of 5.5%) not meeting the clinical requirement (**A1**). The same is illustrated for another session of the same patient in which the delineations differed but the clinical requirement for target coverage of the $CTV_{elective}$ was met (**A2**). A session from another patient in which the $CTV_{elective}$ coverage would not have been met (difference in target coverage of 0.6%) if the AI delineation was used (**B**).*

The figure above illustrates the bladder delineation proposed by the AI network in comparison with the manually adjusted delineation used in the clinic for patient 6 and 7 (see also Fig. 4). In these patients the AI delineation of the bladder would have led to under dosage of $CTV_{elective}$. Patient 6 was a patient in which a diventriculum was present, apparently not being included as bladder by the AI network as pointed out by the red arrows (A1). In another session of the same patient the AI network did include a part of the diventriculum leading to a higher $CTV_{elective}$ coverage meeting the clinical requirement (A2). For patient 7 the differences in target coverage of the AI bladder and the clinical bladder were smaller compared to patient 6 (B).
