# Supplementary material for: Minimizing human interference in an online fully automated daily adaptive radiotherapy workflow for bladder cancer
Source: Radiat Oncol. 2024 Oct 7;19:138. doi: 10.1186/s13014-024-02526-2 (PMC11457325; doi:10.1186/s13014-024-02526-2)
Supplement: Supplementary file 5 — Additional file 5: Target coverage with automated software delineation versus bladder volume differences [file 13014_2024_2526_MOESM5_ESM.pdf]

Target coverage with automated delineation & bladder volume differences

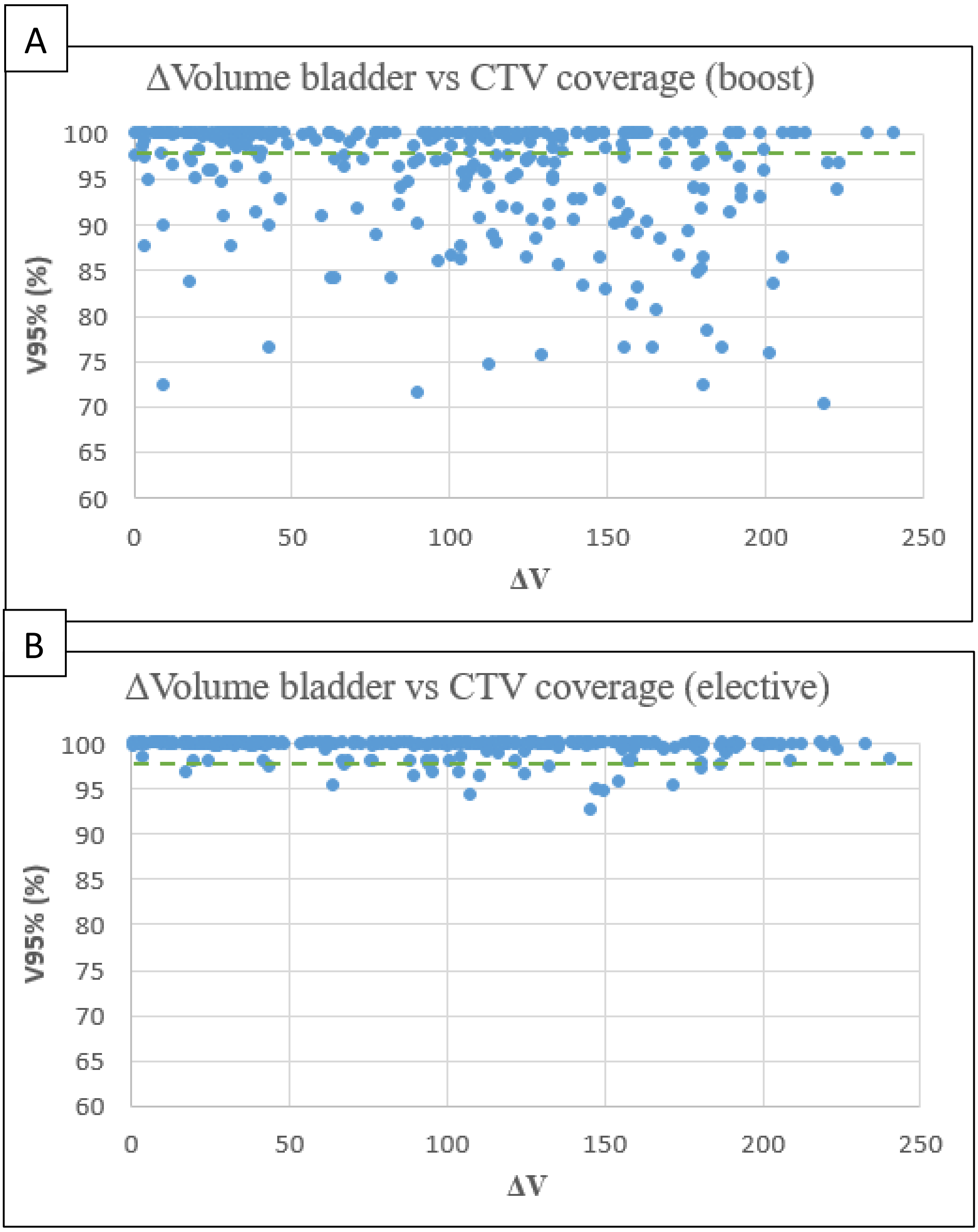

*Additional file 5: Difference in volume between the bladder on the reference CT and the daily CBCT versus the CTV coverage met by the automatic software delineation of targets receiving 40 Gy (**A**) and 55 Gy (**B**). The green dotted line represent the clinical requirement of V95%≥98%.*

Additional file 5. Minimizing human interference in an online fully automated daily adaptive radiotherapy workflow for bladder cancer. Sana Azzarouali, Karin Goudschaal, Jorrit Visser, Laurien Daniëls, Arjan Bel, Duncan den Boer.
